# Consumer Interest in Alternative Grains, Especially Sorghum: A Qualitative Investigation

**DOI:** 10.3390/foods14223918

**Published:** 2025-11-17

**Authors:** Edgar Chambers, Edgar Chambers

**Affiliations:** Center for Sensory Analysis and Consumer Behavior, Kansas State University, Manhattan, KS 66506, USA

**Keywords:** sorghum, grain, consumer, marketing, focus group, qualitative

## Abstract

The market for niche and novelty products in the food industry is growing. However, breaking into the market is tough and reaching the modern-day consumer is harder than ever before. Sorghum, a so-called “ancient grain,” has a chance to be able to compete, but its introduction to human food products needs to be done properly if it is to become a mainstream ingredient. Ten focus groups, with at least two in each of the United States (U.S.) census regions, were conducted to better understand the perceptions, opinions, beliefs, and attitudes for introducing new grain products to the market and specifically regarding grain sorghum. Participants were unfamiliar with sorghum. The few who had heard of sorghum before had difficulty recalling anything about it. When shown a fact sheet on a “new grain” and the nutrition information about the grain, the majority of responses were quite positive. Most consumers were interested in trying products made with the grain and provided various ways the grain (i.e., sorghum) could be introduced to consumers in the United States. A number of terms with potential positive connotations were mentioned by participants that could be used in future research to determine the specific marketability of the grain as an ingredient or in finished products. This qualitative market research demonstrates the open slate that groups such as sorghum commissions, industry, government, and consumer groups have related to sorghum use for consumer food products. It is important to give manufacturers ideas on how best to introduce this “new” grain and to determine which products people would like to see it in. It also is important to understand the marketing options people want to see for a food they have not tried before. Finally, what food advertising options consumers say they pay attention to and which they tend not to focus on is a focus of this research.

## 1. Introduction

The global grain market is undergoing a transformation. Once dominated by wheat, rice, and corn, it now includes a diverse array of ancient and specialty grains such as quinoa, teff, millet, and sorghum. So-called “ancient” grains are defined as grains that have remained largely unchanged by selective breeding over the last several hundred years. The projected compound annual category growth rate of ancient grains is over 38% with more than 75% of ancient grain use as human food in 2024 [1]. This shift reflects evolving consumer priorities regarding health, sustainability, and culinary exploration [2]. Despite growing awareness, a significant portion of consumers remain unfamiliar with many ancient grains beyond quinoa [3]. Marketing efforts must, therefore, be highly educational. Providing recipes, cooking instructions, and information about their nutritional benefits and culinary versatility can help overcome consumer unfamiliarity and encourage trial [4]. The development of innovative food products—such as millet-based porridges, sorghum-infused snacks, and ancient grain pastas—makes these grains more accessible and easier to incorporate into modern diets [5,6].

The increasing popularity of ancient grains is propelled by several interconnected consumer trends: nutrition and health, sustainability, and authenticity. The rise in celiac disease diagnoses and a growing number of individuals with gluten sensitivities have created a substantial market for naturally gluten-free alternatives [5,6]. Sorghum, teff, and millet are naturally gluten-free, making them ideal substitutes for wheat in a variety of products, including flour, pasta, and baked goods. Consumers also are actively seeking foods with superior nutritional profiles, and ancient grains often are perceived as “less refined” and “more digestible” [4,7]. Studies have highlighted the high content of protein, fiber, vitamins, and antioxidants in these grains, which are linked to various health benefits, including improved digestion and weight management [2,6].

Environmentally conscious consumers are increasingly drawn to products that align with their values [8]. Many ancient grains, including sorghum, are known for their resilience, requiring less water and fewer agrochemicals than modern counterparts. The historical and cultural narratives associated with ancient grains add a layer of authenticity that may be a powerful marketing tool [9]. For instance, marketing materials often leverage the “story” of the grain to create an emotional connection with consumers.

Sorghum (*Sorghum bicolor*), a resilient and drought-tolerant crop, has a long history as a staple food in many parts of Africa and Asia, but its commercial use in Western countries has been predominantly for animal feed and biofuels [10]. Sorghum, the fifth-most-produced cereal grain globally, is a particularly promising ancient grain [11].

As a grain, sorghum is praised for its high nutritional value [12]. The high fiber and unique starch structure of sorghum contribute to a lower glycemic index compared to other cereal grains [13]. The slowly digestible starches and resistant starches in sorghum can reduce post-prandial blood glucose spikes, making it a beneficial food for individuals with or at risk of type 2 diabetes [14]. The phytochemicals and dietary fiber in ancient grains, including sorghum, have been shown to help manage cholesterol levels. Studies in animal models and human trials have demonstrated that sorghum consumption can significantly reduce “bad” (non-HDL) cholesterol [15]. The resistant starch and non-starch polysaccharides in ancient grains function as prebiotics, promoting the growth of beneficial gut bacteria. A healthy gut microbiome is crucial for digestive health, immune function, and overall well-being.

Sorghum can be prepared similarly to rice or quinoa in salads, side dishes, and bowls. Its firm, nutty texture holds up well to a variety of cooking methods. Sorghum flour has become a key ingredient in gluten-free flour blends for breads, muffins, cookies, and other baked goods [16,17,18,19,20,21,22]. Research has shown some potential for the use of sorghum and other grains in traditional bread products [23,24]. While the absence of gluten can affect dough elasticity, incorporating other ingredients like starches and hydrocolloids can help overcome these technological challenges and produce high-quality, acceptable products [25,26].

Effective marketing of ancient grains, and particularly sorghum, relies on communicating their unique attributes and benefits. Research has shown that consumers’ purchasing decisions are influenced by a hierarchy of factors, with product origin and price being primary drivers, but with an increasing valuation for flour type and other intrinsic qualities among more knowledgeable consumers [7,27]. Current trends suggest that consumers are focusing on more specific “ethnic” or international flavors instead of broad flavor categories [28].

A key strategy is to market ancient grains as “storied food,” using evocative prose and narratives to elevate the product from a simple commodity to an experience with complex emotional and cultural dimensions [9]. This involves highlighting their ancient heritage, the traditional farming practices used to cultivate them, and their place in cultural foodways. Social media and digital marketing are instrumental in deploying these narratives and fostering a sense of community around the products.

While demand is rising, the ancient grain sector and especially high production grains such as sorghum, face several supply chain and market challenges. It is essential that the infrastructure for sorghum be such that farm production, harvest, and markets are established to reduce market volatility for success [29].

The market for sorghum can be a compelling case study in how a shift in consumer values can transform agricultural commodities into high-value niche products. The success of this sector will not solely be due to the intrinsic health benefits of these crops but is heavily dependent on developing acceptable products and marketing strategies that effectively communicate their unique value proposition. However, there is little research examining the market potential for sorghum or other ancient grains from a wide range of consumers in any Western country. A recent study with college students showed that some products, such as sorghum shrimp grits (grits are a thick porridge-type food usually made with corn), and spiced sorghum cookies, scored highly in acceptability studies with a set of US college students [30]. The rise of alternative grains such as sorghum is expected to continue, in part fueled by AI-driven food system research, the need for clean-label demands, and global flavor exploration [31,32,33]. However, those studies did not specifically mention sorghum. Sorghum and other ancient grains are not just ingredients; they represent a broader philosophy of eating that prioritizes health, heritage, and environmental stewardship.

However, the narrative for sorghum, including the terms and phrases that inspire consumers to adopt a new product or ingredient, is yet to be written. In addition, product developers need to understand what information is critical in order to understand the attributes that must be present in new products [34]. Thus, the objectives of this research were to determine the aspects of sorghum that are meaningful to consumers and can potentially be used to develop products and marketing initiatives for the use of sorghum in particular, and ancient grains more broadly, in successful human foods.

## 2. Materials and Methods

Many potential sensory/consumer research strategies could be used for this research (see, for example, [35,36]). Because the purpose of this research was exploratory, the focus group qualitative research technique was used [37,38,39]. This study was approved by the Institutional Review Board of Kansas State University, proposal number 8492.

### 2.1. Focus Groups and Consumers

A total of 10 focus groups with six to eight consumers in each group were conducted to ensure coverage of the four US census regions (northeast, south, midwest, west). The focus groups were approximately 1.5 h each and were moderated by a professional trained by the Riva Training Institute™ (Rockville, MD, USA). The focus groups were conducted online using consumers recruited from the extended metropolitan areas of Chicago IL, Dallas TX, Los Angeles CA, and White Plains NY. Although originally planned as eight focus groups, differences in participation resulted in the addition of an extra group of consumers in the greater Chicago metropolitan area and an extra group that included consumers from both the Dallas and Los Angeles areas to ensure a more even distribution of participants. Participants were recruited by a professional agency (Peryam & Kroll Research Corporation, Chicago, IL, USA), and were screened to ensure (a) they were between the ages of 18 and 65 years old, (b) had a gender distribution that was approximately 50% male and 50% female, (c) regularly consumed grain-based food products, (d) did at least 40% of the food shopping, (e) had not worked or lived on a farm, (f) did not have a job in marketing research or related areas, (g) were articulate, engaged, and thoughtful, and (h) were available for the full 90 min at the time and date of the focus groups. Participants were paid a stipend by the recruiting agency.

A total of 62 participants in the ten focus groups and the final demographics were 23–64 years old, and 48% male and 52%. Although we did not recruit based on ethnicity, the overall makeup of the consumers tested was 58% Caucasian, 20% African American, 18% Hispanic and 5% Asian.

The focus groups were conducted online to facilitate the collection of data by a single focus group moderator across all groups. Although traditionally conducted in-person, focus groups have recently been conducted online to provide a wider range of participation, reduce costs, provide faster market data, reduce dropout rates, and provide slightly more anonymous participation [40,41]. In addition, online focus groups have been shown to be valid [39,40] and have been used for other recent studies on food [41,42]. It must be noted that with online focus groups, it is more difficult to see nonverbal cues and participant engagement can suffer if moderators do not have specialized training for conducting online groups [42].

### 2.2. Focus Group Structure

**Figure 1:**
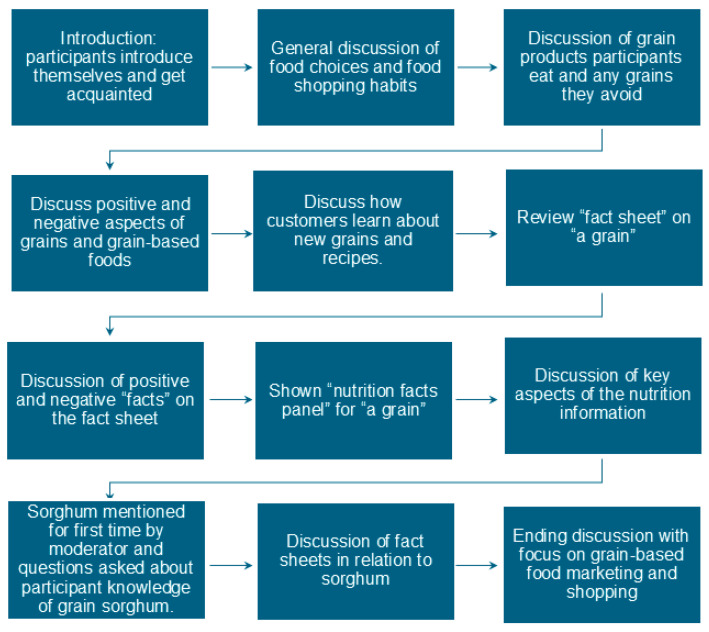
Structure of Focus Groups.

Each group began by making sure all consumers agreed to participate in the research project with Kansas State University; verbal informed consent was obtained. As shown in Figure 1, participants then went through a quick introduction to get acquainted with each other and acclimated to talking online. They were asked to state their first or nickname and to name their favorite food and why it was their favorite. The group then shifted towards general food questions to see why they choose to eat what they eat and to discuss their general food habits. After that, participants were asked specifically about what grains they like to eat and why they choose or avoid choosing certain grains at the store. The group then progressed to gathering terms or phrases, both positive and negative, that the consumers associated with grains, whole grains, and grain-based foods. Consumers were then asked to look at a fact sheet that contained some facts about a “new grain”; they were not aware that we were talking about sorghum at this time. The fact sheet was a combination of items selected from the United Sorghum website (https://www.sorghumcheckoff.com/) and a prior study on sorghum marketing [27]. The fact sheet about the unnamed “new grain”, included a number of aspects of the grain, such as it being an ancient grain, being drought-tolerant, gluten-free, a good source of various nutrients, its use for ethanol fuel, and aspects of sustainability. Consumers were asked to identify aspects that stood out the most to them, either positive or negative. Respondents then did the same with a nutrition label for ½ cup of the uncooked grain. Next, participants were asked questions directly about sorghum, the first time Sorghum was mentioned by the moderator in the focus group. A few questions about sustainability in general and sustainability as it relates to sorghum were included. A discussion was then held about marketing sorghum or a “new grain” and how best to advertise Sorghum or a “new grain” to consumers. Finally, to end the session we asked them to mention one last grain-based shopping habit they had.

### 2.3. Coding and Analysis of Focus Group Data

**Figure 2:**
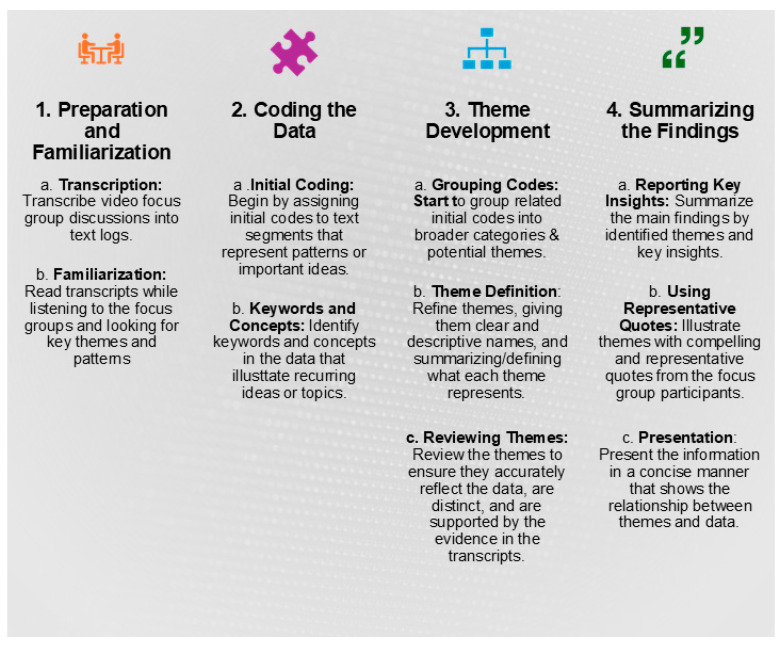
Analysis of Focus Group Data.

Figure 2 shows the process of data analysis used for this research, which is generally described below. The analysis follows typical steps identified in other publications [43,44].

The focus group moderator and researchers reviewed the transcribed focus groups and tapes and began to code specific comments. A list of potential “themes” was developed, and each focus group was replayed and checked against the codes. Various individual codes were then classified into themes, and the data were again reviewed to ensure themes were properly named and specific comments classified appropriately. The themes were developed into key concepts and were summarized. Similar procedures have been used in other types of qualitative research, such as observational research and in-home studies [45,46].

## 3. Results and Discussion

### 3.1. Generalized Food Habits

Most participants claimed they purchased food because it was healthy, it was inexpensive, and a few said they purchased food because it was convenient. A smaller number of participants said that they bought certain foods because they liked the taste. However, when probed further, consumers often stated they considered “tasting good” a prerequisite for considering whether they would purchase most foods. Thus, because it was essential, not optional, it wasn’t initially mentioned as a factor. The sensory aspects of food are critical to acceptance [34]. Taste also was mentioned as a limiting factor throughout the discussion. For example, when discussing grains specifically, consumers often stated they avoided certain grains or grain-based foods because of a flavor or texture issue.

They also described the terms “healthy” and “convenient” as meaning many different things. For example, on what they considered as part of health, people focused more on issues such as organic or types of ingredients rather than nutrition. Participants stated, “I am trying to eat more organic” or “I’ve stopped eating things I can’t pronounce”, for instance. Health also was described in terms of the level of processing associated with food, such as “healthy food is not as processed as unhealthy foods”. These comments may be related to various food classification systems [47] including the increasingly controversial NOVA classification system [48] that has been criticized for oversimplification and classifying many “highly processed” foods as unhealthy, even when they have high nutritional value. They may also be related to the concept that terms like “natural” have little definition and consumers show varying understanding of what foods are natural or not [49,50].

Similarly, convenience was described as different to different people. For some consumers convenience was how far they have to go to shop or how many ingredients they need to make the food, while for others it meant how long it takes to cook. A recent review by Bogard et al. [51] highlights the differences in the conceptualization of convenience.

### 3.2. Grains and Grain-Based Foods

The grains that people said they most commonly ate other than wheat were rice, oats, quinoa, and couscous. Several participants mentioned corn, barley and/or wild rice, although most participants seemed surprised when corn was brought up as a “grain”. Millet, fennel, rye, and flaxseed were sporadically mentioned in a few focus groups. No one mentioned sorghum as a grain they ate. One reason that most of the commonly mentioned grains were named by consumers is that they ate those grains “whole”, “as is” or “minimally processed” as opposed to ingredients in foods other than bread. In contrast, grains such as barley and rye tended to be ingredients in other foods. Because corn (i.e., “sweet corn”) frequently is depicted as a vegetable in the US, this resulted in confusion among some participants when mentioned as a grain by other consumers. In addition, the use of cornmeal, a traditional use of corn as a grain is less common outside the Southern US and Hispanic populations.

In most groups, rice was mentioned as a grain and there was discussion about white, brown, and so-called wild rice (not a true rice). Participants chose among those options depending both on “taste” and perceived healthfulness (e.g., “I choose brown rice because it is a little more healthy than white rice”).

Perceived “unhealthiness” was the most common reason other than “taste” that people would avoid a grain. Statements such as “white bread is not as healthy as whole wheat bread”, “I try to look for whole grain options”, and “I know people who can’t have gluten” were mentioned. The search for more healthful processing of traditional grains and of new healthful grain options is becoming more common in both the US and around the world [52,53,54,55].

### 3.3. Common Associations with Grains

Many people had “positive” associations with grain that were repeated regularly in the focus groups with words such as healthy, natural, pure, and fiber. For example, “Makes you go to the bathroom, but in a good way, because it has fiber.”.

There were a few sporadic mentions of negative connotations with the word “grain” from both a health and sensory standpoint. Terms like “carbs” or carbohydrates were mentioned in a negative way. Previous authors have noted the importance of addressing the overarching concern that some consumers have about grains and other carbohydrate-based foods being an unhealthy part of the diet [52]. From a sensory standpoint, consumers said that waxy, starchy, or gritty were negative connotations associated with some grain products that kept them from choosing those products.

Whole grain, on the other hand, only stood out in a positive way with people commonly saying variations of “Now, whole grain to me, that’s better.” With the term “whole grain”, people kept all of the positive associations that they had with grain but eliminated many of the negative health connotations. According to participants “whole grain” is healthier than regular grain because it has less processing, more fiber, or more vitamins and minerals. The only negative thought expressed towards whole grain was related to “taste”. One individual stated whole grain “sounded less appetizing” because of potential sensory issues they or family members had about whole grain products. Family influences have been noted in prior research as an influencer of whole grain consumption [52].

### 3.4. Fact Sheet and Nutrition Sheet

The fact sheets shown to people had a list of facts about sorghum without telling them it was sorghum, only that it was a new grain. This list included things that would not necessarily be found on a nutrition label but might be used for advertising purposes. Topics such as nutrition, fiber, antioxidants, drought tolerance, water sustainability, ancient grain, animal feed, ethanol production, non-GMO (genetically modified organism) and other aspects were covered.

The only aspect that was perceived as negative by more than one person was the fact that sorghum could be used for ethanol fuel. Comments such as “That doesn’t make you feel like this is going to be something I want to put in my body” or “Even if it’s high in antioxidants, if it can be used for fuel, chances are it’s probably not a good texture you know, it just conjures up a lot of negativity.” A few people said it was OK if it could be used for fuel, but most were vocally opposed to a food that could be used as fuel.

The implication that the grain could be used as animal feed was a direct contrast to it being a fuel. Most consumers liked the idea that it was a multipurpose product that could be used for animal feed as well as human food. Consumers mentioned that it was great that there were additional feed uses for the product.

The rest of the statements were received well. People were especially excited about antioxidants, drought tolerance, that it can be popped like popcorn, and that it may have a long shelf life once processed or cooked. There was some discussion on how long a long self-life is, ranging from 3 days to a month after being cooked. If such a shelf life is possible, it made people comfortable about there not being preservatives in the food.

The nutrition profile, on the other hand, had both positives and negatives associated with it. People were disappointed in the number of calories and carbohydrates the half cup of uncooked Sorghum had. One person stated, “It’s a high amount of calories for a half a cup of anything.” People were much more positive about the vitamins and micronutrients that could be found in sorghum. A number of people liked the vitamin content because they saw it as part of a replacement for taking vitamin supplements and instead getting nutrients naturally through food, with one person stating, “Most people are now taking vitamin supplements, this is a way to get off them and replace it naturally”. Research has shown mixed results related to obtaining nutrients through supplements or food sources, with some suggesting that supplements add little value, while others show a positive health relationship for both food and supplement use to obtain nutrients [56,57,58,59].

Similarly, people were excited about the micronutrients in sorghum because they often don’t see those associated with products. It must be noted, however, that many consumers did not know what micronutrients do for the body, but still viewed them favorably. It was suggested by a couple of participants that the packaging or other advertising should have a statement on what those particular micronutrients do for human health. The use of information about micronutrients can provide both a boost to knowledge but also may provide a “health halo” effect for the overall healthfulness of a food or other particular health aspects [60,61]. It is important to understand the impacts of advertising and the legal ramifications of advertising for specific nutrients or a discussion of specific health benefits or potential disease states [62,63,64].

### 3.5. Sorghum Knowledge and Sustainability

**Figure 3:**
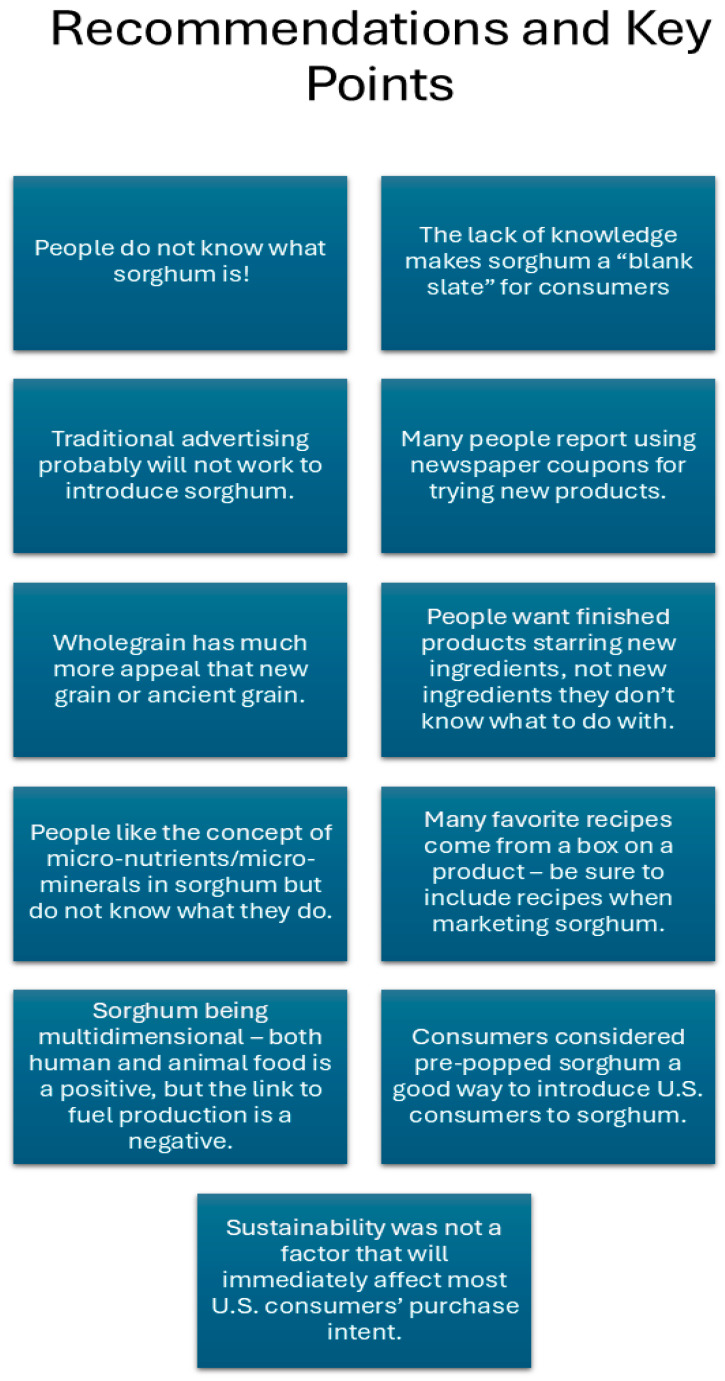
Recommendations and key points for introducing sorghum to the U.S. as an alternative grain for human food.

In this study, only a small number of people had even heard of or had a vague knowledge of sorghum (Figure 3). A couple of those who had heard of sorghum only remembered it from a TV show. “I think I saw it on the side of a barn in the Walking Dead” was stated by one participant. Others had heard about it from a cooking show, from family who owned a farm, and another who did not remember. No one recalled seeing it advertised in a store or had ever used it cooking. Only one person mentioned having seen it as a food ingredient, and no one had eaten sorghum as a food or ingredient. This lack of knowledge can be both a benefit or a disadvantage. Novel foods and other products must “breakthrough” a cluttered market. Strategies for helping novel products succeed such as relating them to existing products but differentiating them enough to promote trial is key [65,66,67,68]. It is critical that consumers overcome the risk associated with unknown products [69] including wasting money on products they may not like or trusting that the advertised benefits are correct.

Sustainability was not a factor for most participants. Although many people did not care much about sustainability, those that did care, cared a lot about it. When further probed on sustainability, many people thought it was a good idea but noted that it was not one that they would pay extra for. However, several consumers mentioned that they would spend extra time to look for products that were sustainable compared to those that are not. 

### 3.6. Advertising

Promotion of new products based on highlighting consumer needs is one of the critical aspects of new products [65,66]. When the group considered how sorghum could be advertised and promoted, many different ideas were mentioned (Figure 1). Interestingly, only a few people liked the idea of traditional advertising on TV or online, perhaps because of advertising clutter [67,68]. The most popular ideas suggested by participants to entice people to try sorghum grain and sorghum grain-based products were (a) coupons issued for products containing sorghum, (b) recipes on the box if someone was to sell whole grain sorghum, and (c) recipe “minutes” and “GIFs” in various media. “If it’s something new, I’m all about something like in-store promotions or if I have a coupon of some sort.”

A large majority of people in the focus groups said they read through coupons sent out by manufacturers or in grocery store ads and reported frequent use of coupons found in the newspaper. Although consumers in these focus groups specifically mentioned preferring a physical coupon, research has provided mixed information on whether digital or print coupons are preferred among consumers [69,70,71]. It is likely that there is a segment of consumers for both types and each has advantages and disadvantages [70].

For sorghum sold as a grain rather than an ingredient in finished foods, a large group of respondents wanted recipes on the box because it was a new product they would not know how to use. Comments such as “At least one or two recipes on the box” were common. In addition, respondents also liked the idea of a QR code on the box, which would bring them to a site with recipes and facts about sorghum. It was noted that some people would not follow through with this extra step, but that it was good for those who would. Participants commented that some people would either be too lazy or wouldn’t know how to look up a QR code. “Also, having a little QR code that can take you to a more extensive recipe list is important.” A few people talked about cooking shows but then stated that they tend to watch cooking shows as entertainment or background noise and rarely use actual recipes from those shows. They were much more likely to use recipes from websites such as YouTube or other social media sites. Thus, having sorghum used in recipes on social media, especially video sites, is both critical to awareness and to learning about how to cook with sorghum. Respondents noted they were much more likely to watch and prepare something they saw on a social media feed if it was between 30 s and 2 min long.

Another idea that received some interest in several focus groups was partnering with a meal kit delivery service because they had become popular during COVID-19. When questioned further, many people said they had either continued using a recipe they originally got from a meal kit or had used a recipe on the advice of someone who got it from a food/meal kit.

### 3.7. Ways to Try Sorghum

Participants were most excited about trying sorghum in a ready-made meal at the grocery store deli section or in a local takeout deli-type store. They liked this idea because it was a low-risk approach to trying out something new. If they didn’t like it, they didn’t feel as though they planned other foods around it. It was something quick and simple that required no thought or time and probably would not cost a lot. Participants in early focus groups wished they could see pictures of various products that might be made with sorghum to get a better idea of how it might be used in finished food products. Thus, in later focus groups, a selection of pictures was provided near the end of the focus group when talking about ways in which to try sorghum. Pictures included such products as grain-based goods, granola bars, pasta dishes, and popped sorghum. The consumers were overwhelmingly in favor of trying popped sorghum, saying it was “new and different”. Products such as granola bars and bread received less support because they were happy with the choices already available or believed those sections were already overcrowded with products.

### 3.8. Terms with Positive Connotations for Marketing Sorghum (Or Any New Grain)

**Figure 4:**
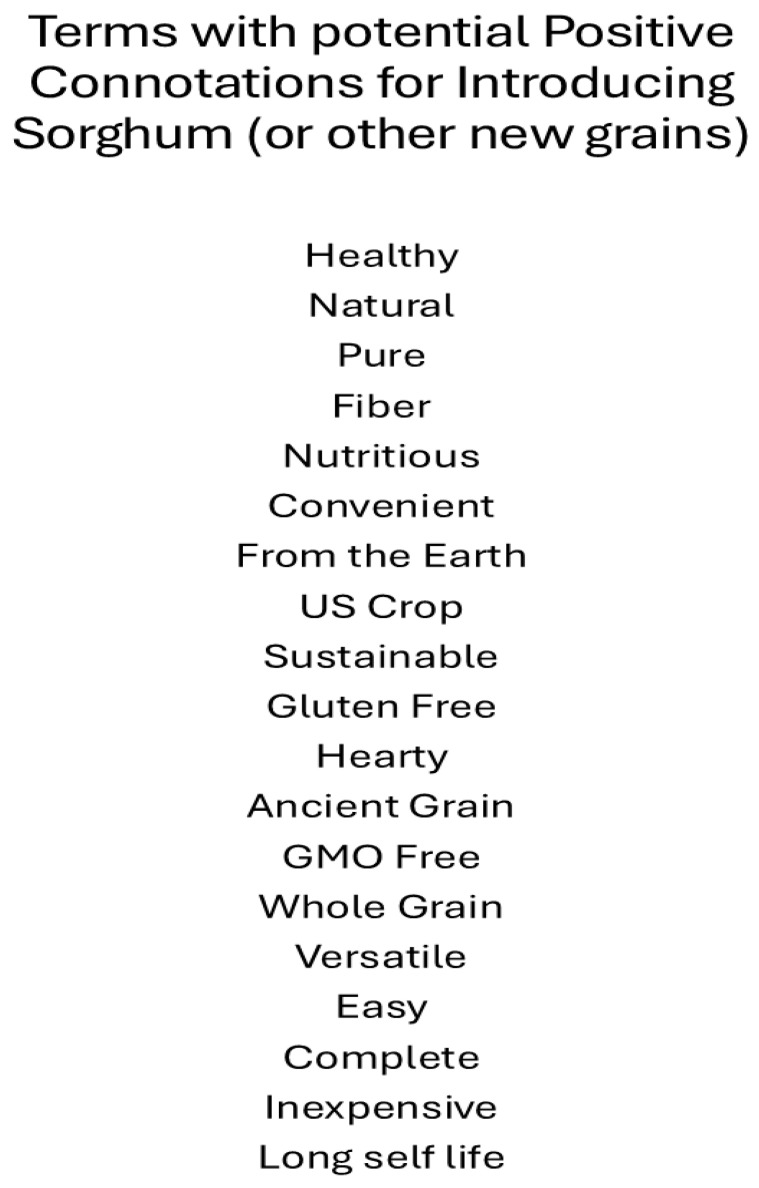
Terms with potential Positive Connotations for Introducing Sorghum (or other new grains).

A number of terms were mentioned that could be used to market sorghum to consumers in the U.S. Positive connotations with attributes and benefits is important when introducing new products [72]. Figure 4 highlights a number of terms that were mentioned by consumers in this research. Some terms were not embraced by more than a few consumers, while others were noted by most consumers as being positive. Regardless, these terms form the basis of future research that should be conducted to determine in sorghum, as a novel food in the U.S. could be marketed successfully.

## 4. Limitations

As with any research, this study is limited in its findings by the characteristics of the consumers studied in the focus groups. Consumers represented many different ages, both genders, various ethnic groups, and all major geographic areas of the US. However, participation was limited to consumers who were available at the times for the study and were part of the database of the supplier. Although this data might apply to other countries, it must be noted that the research was conducted only with U.S. consumers, and use of and motivations for eating a grain such as sorghum will differ in other countries. It is known, for example, that sustainability is more important to consumers in some countries than in other countries [71]. A further limitation of this study is that it was conducted with a general population of consumers in order to highlight the use of alternative grains, in particular sorghum, for the broad population of consumers. Had we chosen a more focused population, such as gluten-intolerant individuals or consumers focused mostly on health, different comments and alternative marketing strategies may have been found.

## 5. Conclusions

The evidence from this and other research underscores the potential of sorghum and other ancient grains for human food use in the U.S. Availability, rich nutritional profiles, and the consumer demand for healthy, sustainable, and naturally gluten-free foods continue to grow. So-called “ancient grains” such as sorghum are positioned to play a critical role in diversifying our food supply and improving public health. Continued research, particularly in product development, marketing, and human clinical trials, is needed to further substantiate the potential of sorghum for use in a wide range of food products in the United States.

The data highlight important marketing and product development opportunities for novel grains, specifically sorghum, by emphasizing consumer preference for “whole grain” messaging due to its positive associations with health, fiber, and minimal processing, which may mitigate the negative “carb” or sensory (waxy, gritty) connotations of grains. For product development, manufacturers should focus on novel, low-risk formats to generate trial, such as popped sorghum and prepared deli-style meals, which were overwhelmingly favored in this study as compared to crowded categories like bread and granola bars. Product developers should also minimize caloric density to address concerns about high calories and carbohydrates. In terms of marketing, direct advertising should focus on positive facts such as antioxidants and the potential to naturally replace supplements with grains that have specific health benefits by explaining the benefits of micronutrients. Promotion should prioritize practical, high-value incentives such as coupons, on-package recipes (with QR codes for more recipes), and short-form social media video recipes to quickly overcome the current profound lack of awareness of sorghum and drive trial of this ancient grain.

## Data Availability

The original contributions presented in this study are included in the article. Further inquiries can be directed to the corresponding author.
